# Peer Review of “Effects of Interventions for the Prevention and Management of Maternal Anemia in the Advent of the COVID-19 Pandemic: Systematic Review and Meta-Analysis”

**DOI:** 10.2196/81699

**Published:** 2025-10-06

**Authors:** Suriya Kumareswaran

**Affiliations:** 1 Johor State Health Department, Johor, Malaysia

**Keywords:** maternal anemia, anemia in pregnancy, COVID-19, pregnancy complications, meta-analysis, maternal and child health, anemia prevention, reproductive health

*This is the peer-review report for “Effects of Interventions for the Prevention and Management of Maternal Anemia in the Advent of the COVID-19 Pandemic: Systematic Review and Meta-Analysis.”*

## Round 1 Review

### General Impressions

The study [1] addresses a crucial public health issue—maternal anemia—and its dynamics in the context of the COVID-19 pandemic. The topic is relevant and timely, particularly given the pandemic’s disruptive effect on health care systems worldwide. However, the manuscript has several shortcomings that require significant revisions for clarity, coherence, and scientific rigor. Below, I provide a detailed assessment with major comments and minor comments for improvement.

### Major Comments

1. Scientific rigor and novelty.

Strength: The focus on maternal anemia interventions during COVID-19 is unique and addresses a significant gap in the literature.

Issue: The study does not establish the novelty of its findings clearly. It cites several similar meta-analyses but does not differentiate its contribution.

Recommendation: Clarify how this meta-analysis advances existing knowledge. Are there new methodologies, expanded datasets, or novel insights?

2. Study design and methodology.

Inclusion criteria: The inclusion of preprints and unpublished data raises concerns about the reliability and quality of the evidence.

Suggestion: Clearly discuss the rationale for including preprints and outline strategies to mitigate biases.

Subgroup analysis: While subgroup analyses are insightful, the interpretation of heterogeneity (*I*²>90% in multiple cases) is not adequately addressed. The sensitivity analyses seem to mitigate this but are not discussed in sufficient depth.

Suggestion: Incorporate a robust discussion of the potential sources of heterogeneity and its implications for the results.

3. Data presentation.

Tables and figures: Tables and figures are overly complex and lack clarity.

Suggestion: Simplify forest and funnel plots for better readability. Ensure that all figures are annotated clearly.

Forest plots: Some rate ratio confidence intervals (eg, in subgroup analysis) overlap with no-effect lines, which undermines conclusions about statistical significance.

Suggestion: Address these overlaps explicitly in the Discussion.

4. Statistical analysis.

Publication bias: The funnel plots indicate substantial publication bias. This is acknowledged but inadequately addressed in the Discussion.

Suggestion: Include a deeper discussion of how this bias impacts the reliability of pooled estimates.

Fixed- versus random-effects models: The rationale for choosing fixed- or random-effects models for different analyses is not well-articulated.

Suggestion: Explain this choice clearly, especially in the context of high heterogeneity.

5. Interpretation of results.

The interpretation of intervention effects (eg, a 17% improvement for iron supplementation) does not account for clinical significance, which may differ from statistical significance.

Suggestion: Discuss the practical implications of these findings, especially in low-resource settings.

6. Language and readability.

The manuscript is riddled with grammatical errors, unclear phrasing, and redundancies. For instance:

“The effect on prevention, control, management and or treatment of anemia was calculated and compared between the intervention and the comparator arms.”

Suggestion: Simplify and clarify language to improve readability.

Acronyms (eg, RR, CI, IFA) are used without clear explanation.

Suggestion: Ensure all acronyms are defined upon first use.

7. Ethical considerations.

The manuscript mentions that some data are unpublished. It is unclear whether these studies adhered to ethical guidelines.

Suggestion: Add a section on ethical considerations, particularly around the inclusion of unpublished studies.

8. Discussion and Conclusion.

Weakness: The Discussion is repetitive and does not critically engage with the limitations of the study or the broader implications of the findings.

Suggestion: Provide a more focused discussion of limitations (eg, high heterogeneity, reliance on observational studies), implications for practice and policy, and recommendations for future research.

### Minor Comments

1. Abstract.

Issue: The abstract lacks precision and overuses vague terms (eg, “several anemia interventions”).

Suggestion: Summarize key findings clearly, avoiding overgeneralizations.

2. Introduction.

The Introduction is overly lengthy and includes redundant information (eg, definitions of anemia repeated multiple times).

Suggestion: Streamline the Introduction to focus on the problem, the gap in knowledge, and the study’s objectives.

3. References.

References are incomplete and inconsistently formatted.

Suggestion: Ensure all references follow a standardized format (eg, APA, AMA).

4. Figures.

Figures are not numbered or titled appropriately.

Suggestion: Include clear figure numbers, titles, and legends for all figures.

### Recommendation for Authors

Based on the above assessment, this manuscript requires major revisions. Key issues include addressing heterogeneity and publication bias in statistical analysis, improving clarity and rigor in data presentation, and enhancing language and readability.
